# Individuals that are consistent in risk-taking benefit during collective foraging

**DOI:** 10.1038/srep33991

**Published:** 2016-09-27

**Authors:** Christos C. Ioannou, Sasha R. X. Dall

**Affiliations:** 1 School of Biological Sciences, University of Bristol , Life Sciences Building, 24 Tyndall Avenue, Bristol BS8 1TQ, UK; 2 Centre for Ecology & Conservation, Biosciences, College of Life & Environmental Sciences, University of Exeter, Daphne du Maurier Building, University of Exeter, Penryn Campus, Penryn, Cornwall, TR10 9FE, UK.

## Abstract

It is well established that living in groups helps animals avoid predation and locate resources, but maintaining a group requires collective coordination, which can be difficult when individuals differ from one another. Personality variation (consistent behavioural differences within a population) is already known to be important in group interactions. Growing evidence suggests that individuals also differ in their consistency, i.e. differing in how variable they are over time, and theoretical models predict that this consistency can be beneficial in social contexts. We used three-spined sticklebacks (*Gasterosteus aculeatus*) to test whether the consistency in, as well as average levels of, risk taking behaviour (i.e. boldness) when individuals were tested alone affects social interactions when fish were retested in groups of 2 and 4. Behavioural consistency, independently of average levels of risk-taking, can be advantageous: more consistent individuals showed higher rates of initiating group movements as leaders, more behavioural coordination by joining others as followers, and greater food consumption. Our results have implications for both group decision making, as groups composed of consistent individuals are more cohesive, and personality traits, as social interactions can have functional consequences for consistency in behaviour and hence the evolution of personality variation.

Living in groups is a widespread adaptation and its independent evolution in a diverse range of taxa suggests that benefits, for example from reduced predation risk^1^, often outweigh costs, such as increased food competition^2^. A new problem then arises that to maintain group cohesion, individuals need to coordinate their behaviour. This can result in the emergence of leaders and followers, hierarchies, or equally-shared (egalitarian) decision making^3^, and has impacts on the payoffs individuals gain from being in a group^4^. Aggregation induces further selection on individuals, including adaptation of morphological^5^, cognitive^6^ and physiological^7^ traits. Socially-mediated influences have also been hypothesised to be important for the evolution and maintenance of another taxonomically widespread behavioural phenomenon, animal personality variation^8^,^9^,^10^. These consistent differences among otherwise similar individuals are evident in a wide range of behaviours, contexts and taxa^11^,^12^, and are known to be important in social hierarchies^13^, group cohesion^14^ and group activity^15^. The strong link between group interactions and personality variation thus require both to be considered to fully understand the evolution and maintenance of either in group-living animals.

Greater consistency over time, contexts or behaviours is symptomatic of stronger personality variation, evident in stronger correlations in behaviour(s). However, such correlations are rarely perfect; in fact, a meta-analysis recently estimated 37% of population-level variation in behaviour across a wide range of species could be attributed to consistent differences among individuals^12^. In other words, the existence of variation between individuals in personality still allows substantial variation around average personality trait values. The remaining variation is due to behavioural plasticity (also known as responsiveness), individual differences in behavioural stability that cannot be accounted for by such plasticity, i.e. ‘unexplained’ within-individual variance, and measurement error^16^,^17^,^18^,^19^. Individuals are known to differ in the extent to which they respond to external and internal stimuli and the extent to which they vary over time after accounting for such plasticity. In other words, individuals vary in both average values for a trait (such as risk taking tendency) and also the variability around these average values (how consistent they are over time); together these inter-individual differences determine personality variation within a population. This consistency can vary with ecological factors, for example predation pressure^20^, suggesting it is itself under selection rather than just average behavioural trait values being selected for. Thus consistency in behaviour seems to be functionally important, either due to the costs of plasticity^18^ or due to some tangible benefit to being consistent and predictable over time.

The evolution of personality variation is of great interest as it represents a reduced flexibility (i.e. plasticity) in behaviour. Performance during social coordination problems, for example, caring for offspring collectively, has been suggested to be a potentially important selection pressure on individuals to both differentiate from their social partners (divide the labour, although see refs 21,22) and be predictable (consistent) while they do so^23^,^24^. Yet this explanation for the evolutionary persistence of such personality variation has only rarely been investigated^25^ and the purported role for consistency per se remains untested. Such coordination problems are likely to feature prominently in many of the contexts that group behaviour is commonly investigated in. For instance, collective foraging by fish in shoals (e.g. leader-follower behaviour from refuges^26^,^27^) requires that individuals remain coordinated with each other to gain protection from predators. However, groups such as fish shoals commonly show fission-fusion dynamics^28^, where frequently changing group membership may not allow individuals to learn the behavioural traits of their groupmates even if their behaviour is consistent over time. In such cases, individuals that are more consistent when alone may be less flexible and responsive to others in a social context, which may make them more likely to adopt leader roles, but be detrimental to group cohesion. In fact, simulations of collective animal behaviour (e.g. refs 4,29) have modelled potential leaders as those with consistent behaviour over time (e.g. travelling in a particular direction) that are also less responsive to the movement of others. It is likely that elucidating the functional consequences of variation among individuals in how much they take risks, as well as how predictably (consistently) they do so, during collective behaviours will be crucial to fully understanding the evolution and maintenance of collective behaviour.

While there is a wealth of evidence for the consequences of average levels of individual behaviour, little empirical effort has been focused on exploring the consequences of differences in within-individual variation (i.e. consistency) that is independent of average levels of expression. Following previous work investigating personality traits using three-spined sticklebacks as a model system, we investigated the role of individual differences in consistency in risk-taking, as well as average levels of risk-taking, in a social foraging experiment. Fish were each tested twice alone, twice in groups of two and twice in groups of four. We estimated individual consistency from the two tests of each fish when tested alone, and used this as an explanatory variable for their behaviour in a social context. We do not attempt to determine the source of differences in consistency between individuals, or whether behaviour is consistent across trials in a social context. Thus our study takes a similar approach to others which have explored the effects of variation in individual behaviour (e.g. average level of boldness) when tested alone on social interactions (e.g. refs 14,15,30,31).

Fish were tested in a V-shaped arena (Fig. 1), with fish starting at the base of the V in a darkened refuge. The latency to leave the refuge was used as a measure of risk-taking tendency as it is widely used to assess boldness, and has been shown to be repeatable in sticklebacks^30^,^32^, correlated to other behavioural measures of risk-taking^33^,^34^ and negatively related to anti-predatory morphological adaptations^35^. This is also ecologically important as refuge use affects predator-prey dynamics^36^, for example by affecting growth rates and fecundity in prey^37^ and having different effects on predators depending on their hunting strategy^38^. The fish had to swim past a visual barrier before being able to see a conspicuous food stimulus in one of two arms at the other end of the arena. When a fish had crossed the arena and entered the arm with the stimulus, two bloodworms per fish were released. Due to the greater analytical tractability in two-fish trials, we focus our analysis of social interactions on these trials, but also explore the consequences of boldness and its consistency on initiating leaving the refuge and food competition in the four-fish trials. Bolder individuals are more likely to accept risk (e.g. from predation^39^,^40^) in return for greater rewards (e.g. during foraging^32^), and have been previously shown to be more likely to lead^30^ and outcompete others for food^2^. After controlling for average levels of boldness, we predicted that more consistent individuals would be less sensitive to being in a social context and thus be more likely to lead and less likely to follow their group mates.

## Results

### Single fish trials

When tested alone, the latency to first leave the refuge and begin exploring the arena was highly correlated in two tests separated by at least 2 days (Spearman’s rank: n = 36, r_s_ = 0.71, P = 1.45 × 10^−6^). It took fish when tested alone a mean of 160.4 seconds to leave the refuge (S.D. = 187.4 s, median = 87 s, range = 5 to 773 s). The mean latency to first leave the refuge in these two trials was used as a measure of an individual’s “boldness”, i.e. their tendency to take risk. Confirming that this measure of boldness was associated with risk-prone behaviour, the time taken to then cross the open arena was shorter in individuals with a greater boldness score (negative binomial Generalised Linear Mixed Model (GLMM), n = 71: χ^2^_1,65_ = 14.9, P = 0.00011, Table S1). The absolute difference between each fish’s latencies to first leave the refuge when tested alone was calculated as a measure of their individual consistency between the two single-fish tests. Differences in individuals’ boldness scores captures inter-individual variation, while the consistency score captures intra-individual variation^16^. Both scales were inverted so that high values corresponded to bold and consistent traits. One explanation for variation in consistencies between fish is the variable number of days between single-fish trials; i.e. fish tested twice in close succession (a minimum of 2 days) may be expected to show more consistency than a greater length of time between tests (a maximum of 6 days). However, there was no evidence that the number of days between tests was correlated with the consistency (n = 36, r_s_ = 0.026, P = 0.88) or boldness scores (n = 36, r_s_ = −0.23, P = 0.18), suggesting these scores were relatively stable over the time scale of the experiment. Additionally, the fish’s standard body length was unrelated to either boldness (n = 36, r_s_ = 0.11, P = 0.52) or their consistency (n = 36, r_s_ = 0.022, P = 0.90). Ensuring that boldness and consistency scores were independent, there was no correlation between these two variables (n = 36, r_s_ = −0.064, P = 0.71).

### Two fish trials: Initiators and leadership

In line with previous work on leadership and animal personalities^2^,^30^, the more bold an individual was relative to the fish it was paired with, the more likely it was to initiate exploration of the arena by being the first to leave the refuge (binomial GLMM, n = 70: χ^2^_1,64_ = 7.34, P = 0.0067). We also found, however, that the relative consistency of a fish also determined whether it was the ‘initiator’, with more consistent individuals (independent of average level of boldness) being significantly more likely to initiate (χ^2^_1,64_ = 4.69, P = 0.030). There were very few cases where the initiator was both less bold and less consistent than their partner (Fig. 2a). As expected if boldness when tested alone generalises to a social context, bolder initiators were quicker to leave the refuge in two-fish trials than initiators assessed as shy when tested alone (Fig. 3; negative binomial GLMM, n = 35: χ^2^_1,28_ = 17.84, P = 2.41 × 10^−5^).

### Two fish trials: Following and cohesion

The time taken between the first (initiator) and second (non-initiator) fish leaving the refuge can be used to measure cohesion and coordination between the two, with initiators only being effective leaders when they are followed^4^,^26^. In two-fish trials, consistent non-initiators followed quickly regardless of the boldness of the initiator, whereas less consistent non-initiators only left soon after the initiator when paired with bold initiators (Fig. 4a; negative binomial GLMM, n = 24: initiator boldness × non-initiator consistency: χ^2^_1,15_ = 4.23, P = 0.040). There was also a significant interaction between the boldness scores of the two fish, with non-initiators with a high boldness score only following bold initiators quickly, while shy non-initiators followed more quickly in general and tended to delay following bold initiators (Fig. 4b; initiator boldness × non-initiator boldness: χ^2^_1,15_ = 5.58, P = 0.018).

We examined whether the non-initiator left the refuge with the initiator also in the refuge (i.e. the initiator returned to the refuge and the non-initiator made its own ‘secondary’ initiation). Despite this only occurring in 7 trials, it was more likely to occur when the non-initiator had a high boldness score (binomial GLMM, n = 35, χ^2^_1,29_ = 3.99, P = 0.046) and the initiator had a low boldness score (χ^2^_1,29_ = 6.35, P = 0.012). Unlike these trends which are somewhat expected, more consistent non-initiators were less likely to later initiate themselves (χ^2^_1,29_ = 7.38, P = 0.0066). This is in direct contrast to the positive effect of consistency on becoming the initiator, suggesting that once the other fish had become the initiator, more predictable fish would not initiate themselves.

### Two fish trials: Functional consequences

We investigated the functional significance of these patterns in social interactions. Initiators ate a greater proportion of the food available (binomial GLMM, n = 70: χ^2^_1,64_ = 8.11, P = 0.0044) and there was a non-significant tendency for initiators to be alone when entering the arm with food (binomial GLMM, n = 54: χ^2^_1,49_ = 3.37, P = 0.066), which would expose them to greater potential risk. Initiators could, however, return to the refuge after first leaving rather than cross the arena, and those that did so were more likely to later cross the arena with the other fish also out of the refuge (binomial GLMM, n = 33: χ^2^_1,29_ = 5.57, P = 0.018) but ate significantly less food (binomial GLMM, n = 35: χ^2^_1,30_ = 4.94, P = 0.026). These trends support the well-established trade-off between risk and reward when individuals forage collectively^41^,^42^,^43^, and as expected, fish that reached the food arm alone ate a significantly greater proportion of the food available (binomial GLMM, n = 56: χ^2^_1,50_ = 8.36, P = 0.0038).

Linking this functional perspective back to inter-individual variation, the proportion of bloodworms eaten by a fish significantly increased the more bold and consistent it was relative to its partner in two-fish trials (Fig. 5; binomial GLMM, n = 72: relative boldness: χ^2^_1,65_ = 8.27, P = 0.0040; relative consistency: χ^2^_1,65_ = 4.73, P = 0.030). Bolder or more consistent initiators were not more likely to cross the arena on their first trip without returning to the refuge compared to shyer initiators (binomial GLMM, n = 35: boldness: χ^2^_1,29_ = 2.11, P = 0.15, consistency: χ^2^_1,29_ = 0.35, P = 0.56) or to cross alone (binomial GLMM, n = 33: boldness: χ^2^_1,27_ = 0.0004, P = 0.98, consistency: χ^2^_1,27_ = 0.56, P = 0.45), suggesting it may be the effect of how bold and/or consistent they were on whether they initiated that influenced the amount of food eaten. To test this further, the proportion of food eaten was re-analysed with both personality trait score differences and being an initiator as explanatory variables: the only significant influence was whether a fish was the initiator (binomial GLMM, n = 70: χ^2^_1,62_ = 6.11, P = 0.013, when mean boldness main effects were included in the model), with the differences in the personality trait scores of the two fish losing their statistical significance (boldness difference: χ^2^_1,62_ = 3.82, P = 0.051; consistency difference: χ^2^_1,62_ = 2.23, P = 0.14). Thus, both boldness and consistency determine which fish is the initiator, which in turn determines the proportion of food eaten by a fish.

### Four fish trials

In four-fish trials, an effect of consistency was found on the probability a fish was the initiator, although this interacted significantly with the fish’s relative boldness unlike in two fish trials (binomial GLMM, n = 66: χ^2^_1,59_ = 9.89, P = 0.0017). This indicates that the two effects were interdependent, i.e. fish needed to be both bolder and more consistent than their partner to be the initiator (Fig. 2b). However, unlike trials with pairs of fish, there was no indication that relative boldness (binomial GLMM, n = 72: χ^2^_1,65_ = 2.73, P = 0.098) nor consistency (χ^2^_1,65_ = 2.44, P = 0.12) had an effect on the proportion of food eaten. Initiators in these four fish trials consumed a greater proportion of the food available (binomial GLMM, n = 66: χ^2^_1,60_ = 12.24, P = 0.00047), thus personality differences had an indirect effect via determining which fish successfully initiated movement from the refuge.

## Discussion

The importance of variability between individuals in group interactions is a key question in understanding the evolution, maintenance and behaviour of animal groups. Personality variation is of particular interest as, by definition, these inter-individual differences will be persistent over relevant timescales. In addition to inherently social traits such as aggression, behaviour under risk (e.g. boldness, exploration and neophobia) has been shown in a wide range of species to affect interactions within groups and their functioning^2^,^15^,^30^,^31^. Moreover, formal evolutionary models predict that social interactions can themselves select for behavioural differences between individuals through frequency-dependent processes^4^,^44^,^45^,^46^. Factors associated with inter-individual variation in consistency of behaviour have been investigated empirically^20^,^47^ and the benefit of plasticity is clear^44^; the results presented here complement this existing work by now showing that consistency per se can also be advantageous, thus potentially acting as a selective force in the evolution of animal personality variation in social contexts.

Leadership, which can be defined as a disproportionate influence on others by a subset of the group^26^,^29^, is often correlated with bold and active traits^30^,^31^. As leadership allows individuals to have a greater influence on group behaviour, it is often beneficial (although can have costs^48^) and explains why it is widespread in animal groups^49^. We find that more consistent, as well as bolder, individuals were more likely to initiate leaving a refuge, suggesting they have a greater tendency to lead. In two-fish trials, consistent and bold individuals ate more food, with this being mediated via the effect of boldness and consistency on which fish was the initiator. Thus, in the social foraging context investigated here, being consistent can at least partially compensate for shyness. In addition to these trends which are typically associated with boldness and in contrast to our initial predictions, more consistent non-initiators also followed regardless of the boldness of the initiator, and were less likely to attempt their own initiation later in the trial. Cohesion is thus greater in groups composed of consistent individuals. Over longer-term repeated interactions, these effects may result in greater social feedback^30^, where distinct leader and follower roles are more likely to emerge in groups containing consistent individuals. Thus, while plasticity in behaviour may be advantageous^18^,^44^, we show that there can be benefits to behavioural consistency for both leadership and followership.

Although we find numerous consequences of individual consistency for social interactions, further research is needed to determine to what extent the differences in consistency between individuals were due to different responsiveness to changing conditions (i.e. behavioural plasticity^19^) or due to differences in the repeatability of behaviour which cannot be accounted for by this plasticity^16^,^17^,^18^. Both sources of consistency are supported by experimental work^18^,^19^, although quantifying their relative contributions to consistency in behaviour requires many repeated tests of individuals over time^18^. This was not possible here due to the need to also test fish in group trials and to avoid habituation effects, and this was not the main aim of our study which was focused on consequences, rather than causes, of individual consistency in social groups. As with any other trait, we can however rule out that variation between individuals in our measure of consistency was due to ‘random’ noise, such as from measurement error^16^, because this consistency had multiple effects in a social context. In fact, these results suggest that the effect size of consistency is substantial, and that many existing data sets of animal personalities and their consequences could be reanalysed to include the difference in measurements of individuals between testing periods, rather than just the average, so long as individuals are tested more than once to quantify individual differences.

Further work is also needed to determine the mechanism(s) by which consistent individuals are more likely to lead and follow. While some effects of personality in social behaviour are intuitive, such as bolder individuals leading groups, other effects have been documented without a clear underpinning mechanism, for example, why shyer individuals increase following when paired with bolder individuals^30^. Group cohesion, information transfer and group decision making in fish is believed to be mediated mainly through movement^28^, which is relatively quantifiable with automated tracking software (e.g. ref. 50). However, how personality differences between individuals (and variation from other sources such as prior experience) affects fine-scale movements and how these result in group decisions remains unknown, with current work only beginning to use relatively coarse movement parameters such as average speed and tortuosity^26^,^51^.

Previously studies have demonstrated that bolder individuals are able to outcompete shyer fish during foraging^2^. Although this will provide selection pressure for boldness, the finding that more consistent fish showed a similar advantage suggests a selection pressure for consistency in behaviour itself in the collective foraging context of our study. While the benefits to being bold are offset by increased predation risk^39^, consistency may be selected against if it makes individuals more predictable in repeated interactions with the same competitor or predator^17^,^18^, or less sensitive to changes in the environment (i.e. lower adaptive plasticity). As our experiment was conducted under relatively stable conditions, further work is required to address these issues to quantify the costs, as well as the benefits, of behavioural consistency.

## Methods

### Experimental subjects

Fish (40 ± 2.6 mm mean ± S.D. standard body length) were caught from the River Cary, Somerset, UK (grid ref: ST 469 303). Fish were kept in 120 × 45 × 37.5 cm glass tanks. They were fed flake food and defrosted bloodworms ad libitum once per day and were held in the laboratory for at least three months before testing. Lighting was on a 10:14 day:night cycle and water temperature was held at 15–16 °C throughout. Under these conditions, three-spined sticklebacks are not in reproductive condition^52^ and were thus not sexed, following other studies using these fish to examine collective behaviour^5^,^27^,^30^ (sex has also been shown to be unrelated to boldness in this species^2^).

### Experimental protocol

Fish were tested in 3 batches of 12 fish each. Individual tagging followed the procedure of Webster and Laland^53^, and fish were held in three 16.5 × 12.7 × 12.7 cm breeding nets (4 fish per net) positioned in one of the stock tanks. After 4 days to habituate with the tags, each fish was tested once per day with a non-test day in the middle separating two three-day testing periods. Within each testing period, each fish was randomly assigned to a set of 4 fish, and on each day a set was tested as four single fish, two pairs or a single group of 4. The order of these group size treatments was randomised using a Latin square, so that each set of fish and each treatment was tested once per day. There were thus 7 trials per day, conducted in a random order. For the second three-day testing period, fish were again randomly assigned to make up new sets of four fish, with the constraint that a maximum of two fish could be in the same set in the first and second three-day periods. Testing took place between 26.4.13 and 14.6.13.

**Figure 1:**
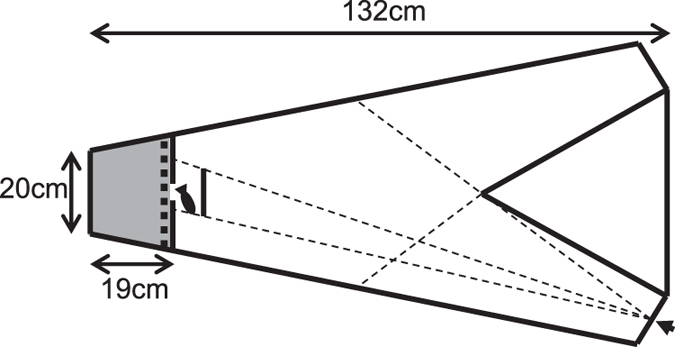
The experimental tank (to scale). Fish were habituated in a mesh-covered refuge (shaded grey) before the door (thick dotted black line) was raised remotely. The fish had to swim past a visual barrier before being able to see a food stimulus in one of two arms at the other end of the arena; the dotted lines represent lines of sight when the food stimulus was placed in the right side arm (bottom right of the figure, indicated by the arrow). The dashed lines connected to the food stimulus represent lines of sight when the fish first leave the refuge, and the other line is the point at which the fish were deemed to have made a decision as at this point the stimulus in the other arm (if present) would not be visible. Once this decision had been made, 2 bloodworms per fish being tested were released.

**Figure 2:**
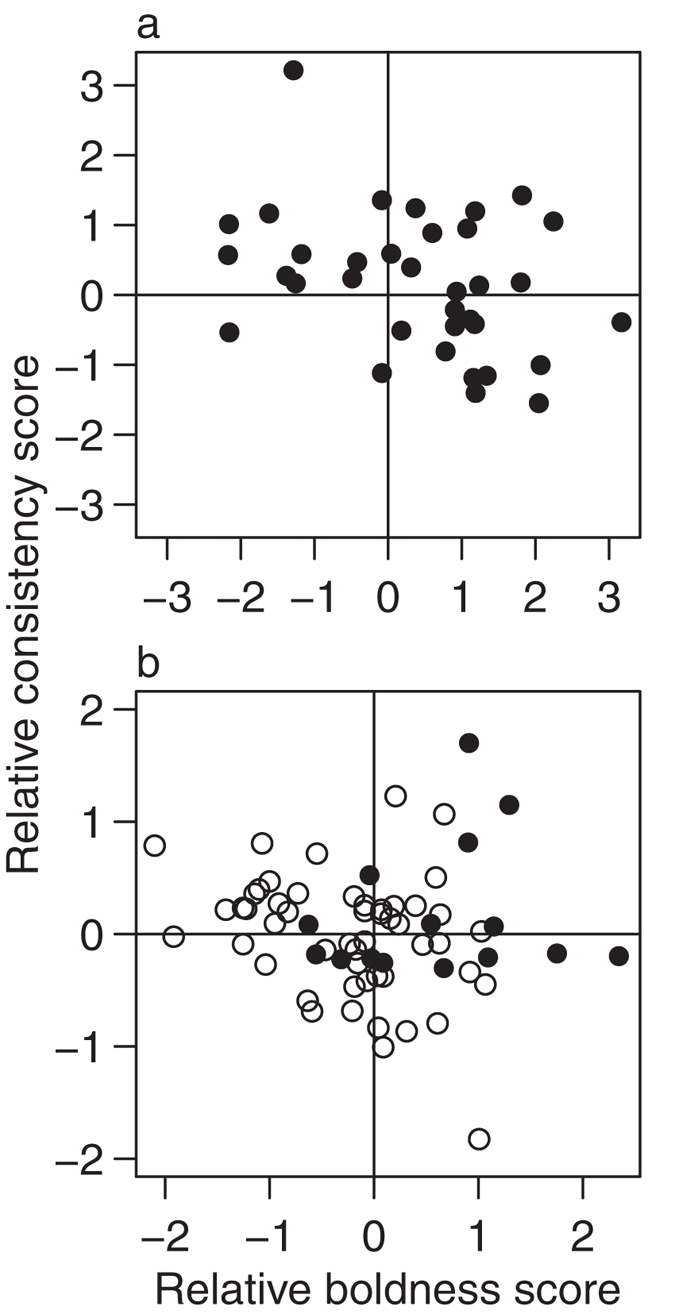
The effect of relative personality trait scores on whether a fish initiated exploration of the arena (i.e. was the first to leave). (**a**) Shows the effect of the difference between the two fish’s scores for each trait in two-fish trials. (**b**) Shows the effect of the difference between a fish’s score in a trait and the median of the group for that trait for groups of 4 fish. Initiators are represented by filled circles and non-initiators by open circles.

**Figure 3:**
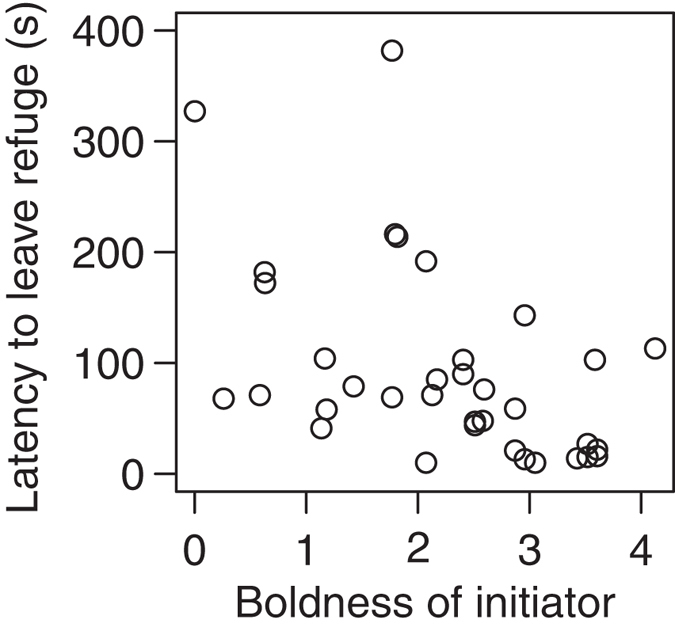
The effect of the initiator’s boldness score on their latency to leave the refuge in trials of two fish.

**Figure 4:**
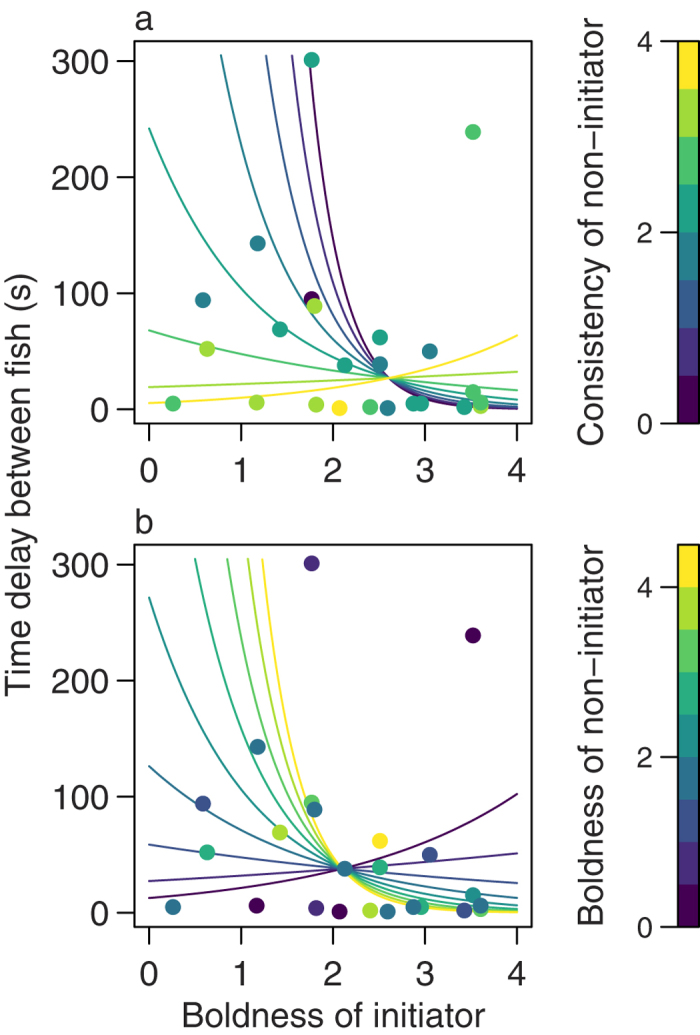
The effect of personality trait scores on following (the time delay between initiators and non-initiators) in two fish trials. Filled circles are observed data. The colours represent the non-initiator’s consistency score (**a**) and non-initiator’s boldness score (**b**), binned every 0.5 units of each score. Coloured lines are fits for each binned interval, calculated from the coefficients of the GLMM which includes the two significant interaction terms (Table S1; note that the models use continuous, not binned, data). The main effect of trial order is fixed at its mean value in the data, as is the value of non-initiator boldness in (**a**), and non-initiator consistency in (**b**).

**Figure 5:**
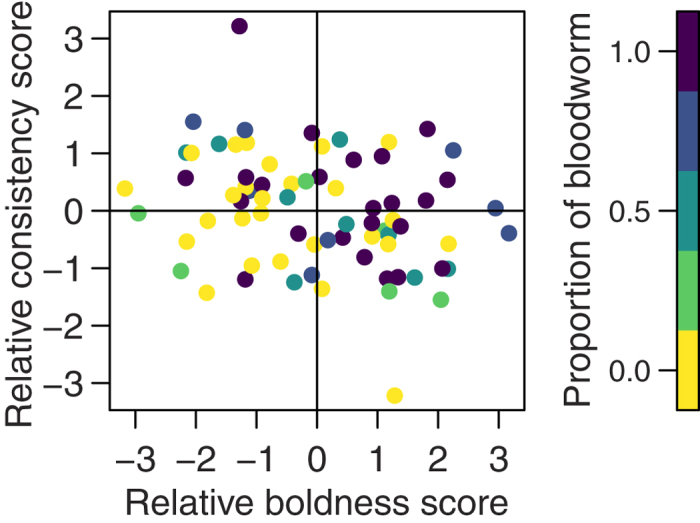
The effect of relative personality trait scores on the proportion of food eaten by each fish in two-fish trials. The proportion of food is represented on a colour scale. Relatively bolder and more consistent fish ate more food (i.e. there are darker points in the top right quarter compared to the bottom left quarter).

Trials were carried out in a white Perspex V-shaped arena (Fig. 1). At the end of one arm, a pipette was held in a white opaque tube so that the food (bloodworms) inside it was not visible; instead red PVC tape was wrapped around the end of the pipette which extended past the end of the tube to provide a standardised 17 × 3 mm (length × diameter) visual stimulus. Red on a white background has been shown previously to be highly conspicuous sticklebacks^54^. The side of the pipette and stimulus was randomised for each trial. An air bubble was positioned at the tip of the pipette to minimise olfactory cues from the food. The water level within the testing tank was 11.5 cm. For each trial, the individual or group to be tested was moved to the refuge area and given 2 minutes to habituate. The door was then gently raised, allowing the fish access to the main arena. Once a fish crossed the arena and the decision line (Fig. 1), two bloodworms per fish were gently pipetted into the tank. If the arm without the stimulus was chosen first (which only occurred in 13 out of 215 trials), the trial continued until the rewarding arm was chosen. Trials were ended once all the bloodworms had been eaten. The time taken to leave the refuge was not censored, which has been shown to be important in estimating individuals’ consistency^16^. Trials were filmed from above using a Panasonic SD800 camcorder at a resolution of 1920 × 1080. Behaviour was scored from these videos, and were analysed blind in terms of the experimenter (CCI) not knowing the behaviour of the test individual(s) in other trials. Testing took place between 09:30 and 15:00. Experimental procedures were approved by the University of Bristol Ethical Review Group (UIN UB/11/042) and were carried out in accordance with institutional guidelines.

### Statistical analysis

Due to the right-skewed distribution typical of latency data, the latency to leave the refuge during single-fish trials was log10 transformed before calculating boldness and consistency scores. In most statistical tests of 2 fish trials, the personality trait scores of refuge use (boldness) and its consistency for each fish, plus the boldness score of the fish’s partner, were used as covariates in GLMMs. Models initially included all two-way interactions between personality trait scores. Trial order (i.e. testing day, 1 to 6) was included throughout as a main effect to account for any habituation/training over the course of the trials. Fish identity was included as a random effect in all models, and was crossed with trial identity as an additional random effect where multiple fish in a trial were included to account for this non-independence.

In the binomial Generalised Linear Mixed Models (GLMMs) used to analyse which fish was the initiator, relative measures of personality scores were used as covariates because the response variable within a trial is mutually exclusive as only one fish could be the initiator. Thus the relative difference between the two fish’s personality trait scores is the important factor. In two-fish trials, this was the difference between the two fish’s boldness scores and the difference in their consistency scores, and in four-fish trials, the difference between each fish’s score and the median of the group for that score (calculated separately for boldness and consistency). This was also the approach used to analyse the proportion of bloodworms eaten within each trial as each bloodworm could be eaten by only one fish.

Details of GLMMs and full statistical results are given in Table S1. All tests were two-tailed with an alpha of 0.05. Non-significant interaction terms were removed from the models. In analyses differentiating between initiators and non-initiators, data were excluded when there were two fish that first left the refuge simultaneously so that which was the initiator could not be distinguished (this occurred in one two-fish trial and three four-fish trials). Sample sizes were reduced further in some analyses when the initiator or non-initiator did not perform a particular behaviour before the end of the trial (e.g. leave the refuge); the reported sample sizes in the Results (n) take these cases into account. R 3.0.2^55^ was used for all analyses.

## Additional Information

**How to cite this article**: Ioannou, C. C. and Dall, S. R. X. Individuals that are consistent in risk-taking benefit during collective foraging. *Sci. Rep*. **6**, 33991; doi: 10.1038/srep33991 (2016).

## Supplementary Material

Supplementary Information

Supplementary Table S1
