# Supplementary material for: Individuals that are consistent in risk-taking benefit during collective foraging
Source: Sci Rep. 2016 Sep 27;6:33991. doi: 10.1038/srep33991 (PMC5037426; doi:10.1038/srep33991)
Supplement: Supplementary Information [file srep33991-s1.pdf]

Individuals that are consistent in risk-taking benefit during collective foraging

Christos C. Ioannou, Sasha R.X. Dall

Table S1: Summaries of the Generalised Linear Mixed Models (GLMMs). Shown are coefficient estimates, their standard errors, their Z score, and the corresponding P value. Tests are presented in the order in which they appear in the main text.

Test and response variable	Explanatory variable(s)	Estimate	S.E.	z value	P value
**SINGLE FISH**					
negative binomial GLMM	Trial order (day 1 to 6)	0.445	0.118	3.77	0.00016
Latency to cross the arena	Boldness	-0.989	0.256	-3.87	0.00011
	Consistency	-0.312	0.358	-0.87	0.38448
**TWO FISH**					
binomial GLMM	Trial order (day 1 to 6)	1.01×10^{-10}	0.156	0	1
Whether a fish is the initiator	Difference in boldness	0.581	0.215	2.71	0.0067
	Difference in consistency	0.640	0.296	2.17	0.0303
negative binomial GLMM	Trial order (day 1 to 6)	-0.2523	0.069	-3.66	0.00026
Time taken for initiator to leave refuge	Initiator boldness	-0.5036	0.1192	-4.22	2.40×10^{-5}
	Initiator consistency	0.0591	0.2092	0.28	0.77754
	Non-initiator boldness	0.2125	0.1216	1.75	0.08057
negative binomial GLMM	Trial order (day 1 to 6)	0.0926	0.163	0.57	0.571
Time delay between the initiator and non-initiator leaving	Initiator boldness	-1.65	1.28	-1.29	0.1976
	Non-initiator consistency	-2.54	0.982	-2.59	0.0097
	Non-initiator boldness	1.53	0.753	2.03	0.0419
	Initiator boldness x Non-initiator consistency	0.973	0.473	2.06	0.0396
	Initiator boldness x Non-initiator boldness	-0.729	0.309	-2.36	0.0181
binomial GLMM	Trial order (day 1 to 6)	-1.884	1.692	-1.113	0.2655
Whether non-initiator left refuge with initiator in refuge	Non-initiator consistency	-40.001	14.725	-2.716	0.0066
	Initiator boldness	-13.582	5.388	-2.521	0.0117
	Non-initiator boldness	11.859	5.936	1.998	0.0458
binomial GLMM	Trial order (day 1 to 6)	0.0113	0.3353	0.03	0.9732
Proportion of food eaten by a fish	Whether the fish was the initiator	4.6833	1.6443	2.85	0.0044
binomial GLMM	Trial order (day 1 to 6)	-0.1458	0.3029	-0.481	0.6303
Whether fish crossed arena alone	Whether the fish was the initiator	2.9638	1.6145	1.836	0.0664
binomial GLMM	Trial order (day 1 to 6)	-0.0958	0.2424	-0.4	0.693
Whether initiator crossed arena alone	Whether the initiator did not return to the refuge	1.9652	0.8326	2.36	0.018

binomial GLMM	Trial order (day 1 to 6)	0.0118	0.3707	0.03	0.975
Proportion of food eaten by initiator	Whether the initiator did not return to the refuge	3.6846	1.6584	2.22	0.026
binomial GLMM	Trial order (day 1 to 6)	0.294	0.236	1.25	0.2131
Proportion of food eaten by a fish	Whether the fish crossed arena alone	2.769	0.958	2.89	0.0038
binomial GLMM	Trial order (day 1 to 6)	-0.0193	0.2844	-0.07	0.946
Proportion of food eaten by a fish	Difference in boldness	1.1031	0.3835	2.88	0.004
	Difference in consistency	1.2189	0.5606	2.17	0.03
binomial GLMM	Trial order (day 1 to 6)	-0.007205	0.228187	-0.032	0.975
Whether the initiator did not return to the refuge	Initiator boldness	0.60568	0.416481	1.454	0.146
	Initiator consistency	0.355286	0.603923	0.588	0.556
	Non-initiator boldness	0.04851	0.445035	0.109	0.913
binomial GLMM	Trial order (day 1 to 6)	-0.06346	0.23656	-0.27	0.79
Whether initiator crossed arena alone	Initiator boldness	-0.00749	0.36464	-0.02	0.98
	Non-initiator boldness	0.26674	0.43002	0.62	0.54
	Initiator consistency	0.45197	0.60273	0.75	0.45
binomial GLMM	Trial order (day 1 to 6)	-0.00891	0.31976	-0.03	0.978
Proportion of food eaten by a fish	Difference in boldness	0.88185	0.45134	1.95	0.051
	Whether the fish was the initiator	3.59371	1.454	2.47	0.013
	Difference in consistency	0.89936	0.60232	1.49	0.135
**FOUR FISH**					
binomial GLMM	Trial order (day 1 to 6)	-0.446	0.445	-1	0.316
Whether a fish is the initiator	Difference in boldness	3.949	1.66	2.38	0.0173
	Difference in consistency	-3.269	1.987	-1.65	0.0999
	Difference in boldness x Difference in consistency	9.713	3.088	3.14	0.0017
binomial GLMM	Trial order (day 1 to 6)	-0.0302	0.1148	-0.26	0.7921
Proportion of food eaten by a fish	Difference in boldness	0.4573	0.2767	1.65	0.0984
	Difference in consistency	-0.6025	0.386	-1.56	0.1185
binomial GLMM	Trial order (day 1 to 6)	-0.0849	0.2119	-0.4	0.68848
Proportion of food eaten by a fish	Whether the fish was the initiator	4.0156	1.1478	3.5	0.00047
